# Supplementary material for: Knowledge, attitudes, and practices (KAP) of nutrition among school teachers in Bangladesh: A cross-sectional study
Source: PLoS One. 2023 Mar 24;18(3):e0283530. doi: 10.1371/journal.pone.0283530 (PMC10038292; doi:10.1371/journal.pone.0283530)
Supplement: Attachment — Submitted filename: Response to reviewers.docx [file pone.0283530.s001.docx]

**Knowledge, Attitudes, and Practices (KAP) of Nutrition among School Teachers in Bangladesh: A Cross-Sectional Study**

**[PONE-D-22-31869]**

I want to express my gratitude to the chief editor and the reviewers for their insightful comments and recommendations that I have already received. It was quite beneficial to consider new ideas in order to enhance the work. Please see below my detailed response to your comments and recommendations. I made an effort to take into account each advice that you so graciously made in light of my expertise and understanding.

**Comments from Reviewer and Author’s responses:**

**Reviewer #1:**

Thanks for the invitation and I am happy reading the paper. I must say this is a well written manuscript the methodology is strong enough however I have some addressable comments.

**1.** The ethical approval of the paper is missing which is the strongest demerit of the paper for being rejected. Please add the approval number of your institution that had been given in this regard.

**Response from Authors:** Thanks to the reviewer for the valuable comment. We added the number of ethical approval (NSTU/SCI/EC/2022/113) given from the institution named Noakhali Science and Technology University, Bangladesh.

**2**. Second: the abstract is not organized and not even the style of PLoS one criteria.

**Response from Authors:** Abstract is organized according to the PLOS ONE style.

**3.** The rationality of the paper poor why this study important is missing in my sense.

**Response from Authors:** Thank you for your valuable comments and suggestions to this work. As per your suggestions the rationality in the background section is updated.

**4.** The generalizability of the paper is poor due to its inherent study design and small sampling size.

**Response from Authors:** Thank you for this comment.

Based on response rate, we were able to keep only 280 school teachers in this study who filled up the questionnaire completely. Furthermore, in context of Bangladesh, the number of school teachers compare to the number of students is low and typically they have much work load. So, a lot of them are reluctant to participate in this study. Therefore, we recommend similar future study with large sample size to evaluate the whole scenario of the country.

**5.** Conclusion is overtly written please be specific based on your findings.

**Response from Authors:** We rewrite the conclusion focusing on our main findings along with the future recommendations as this study is the first study in Bangladesh evaluating teacher’s knowledge regarding nutrition.

**6.** How did you select covariates for final model that is missing.

**Response from Authors:** Thanks for this comment.

Selection of co-variates for final model is done by observing marginal association with the study’s outcome which is determined by a previous pilot study. We also considered the previous literature published in other countries to select the covariates for our study.

**Reviewer#2:**

Well written article. the study highlights the need for the teachers to be given more training. the analysis has been carried out nicely. the discussion and the results are aptly described. the conclusions are crisp.

**Response from Authors:** Thanks to you for your valuable observations, comments and recommendations.
